# Efficacy and Safety of Shi Cervical Rotational Manipulation in Patients With Atlantoaxial Joint Subluxation: Protocol for a Randomized Controlled Trial

**DOI:** 10.2196/57865

**Published:** 2024-08-13

**Authors:** Deng Zhen, Shang Haibin, Ai Youli, Yuan Weian, Wang Huihao, Zhan Hongsheng, Li Guozhong, Ding Ren, Shen Zhibi

**Affiliations:** 1 Department of Orthopedics & Traumatology Shanghai Baoshan Hospital of Integrated Traditional Chinese and Western Medicine Shanghai China; 2 Department of Orthopedics & Traumatology Shuguang Hospital Affiliated to Shanghai University of Traditional Chinese Medicine Shanghai China

**Keywords:** atlantoaxial joint subluxation, Shi cervical rotational manipulation, efficacy, safety, randomized controlled trial

## Abstract

**Background:**

The clinical diagnosis of atlantoaxial joint subluxation (AJS) in traditional Chinese medicine (TCM) is characterized by an unequal distance between the lateral mass of the atlas and the odontoid process on imaging, resulting in neck pain accompanied by symptoms such as dizziness, headache, and limited cervical mobility. In Shanghai, Shi cervical rotational manipulation (SCRM) is a commonly employed TCM manual therapy for treating this condition. Nevertheless, there is a lack of evidence-based medical information regarding the clinical efficacy and safety of this technique.

**Objective:**

The principal aim of this study is to evaluate the efficacy and safety of SCRM in patients diagnosed with AJS.

**Methods:**

This study is a prospective randomized controlled clinical trial that will be conducted at a single center and that has a follow-up period of 24 weeks. A total of 96 patients diagnosed with AJS will be recruited from outpatient and inpatient clinics at Shanghai Baoshan Hospital of Integrated Traditional Chinese and Western Medicine. These patients will be randomly assigned to either the experimental group (SCRM) or the comparison group (basic cervical manipulation [BCM]). Treatment sessions consisting of SCRM or BCM will be administered twice a week for a duration of 4 weeks. Clinical monitoring indicators include the presence or absence of clinical symptoms as recorded on a symptom recording form, cervical imaging examination findings using cervical computed tomography, degree of neck pain measured by a visual analog scale (VAS), cervical range of motion assessed through cervical mobility measurement, degree of vertigo evaluated using the Vertigo Symptoms Scale-Chinese Version (VSS-C), and adverse events that may occur during the follow-up period. The time points for data collection and follow-up are baseline and postintervention (weeks 4, 8, 12, 16, 20, and 24).

**Results:**

This paper presents an overview of the reasoning and structure of a prospective randomized controlled trial with the objective of investigating the clinical efficacy and safety of SCRM in patients with AJS by assessing improvements in clinical symptoms, neck pain severity, and vertigo severity and evaluating changes in cervical imaging findings. Recruitment was started in March 2023. By the end of May 2024, 76 patients were included in this project. The last follow-up data are predicted to be collected by the end of February 2025.

**Conclusions:**

This investigation will yield dependable evidence regarding the efficacy and safety of SCRM in patients with AJS.

**Trial Registration:**

Chinese Clinical Trial Registry ChiCTR2300068510; https://www.chictr.org.cn/showprojEN.html?proj=186883

**International Registered Report Identifier (IRRID):**

DERR1-10.2196/57865

## Introduction

Joint subluxation is a distinct medical diagnosis within the realm of traditional Chinese medicine (TCM) [1]. In November 2020, the National Health Commission of China and the China Administration of Traditional Chinese Medicine officially incorporated the term “joint subluxation” into the updated edition of the “International Classification of Diseases 11th Revision, ICD-11” instead of “Gu Cuo Feng,” which was previously widely used in TCM clinical diagnosis, thereby establishing its recognition as a universally accepted diagnostic term [2]. While joint subluxation can manifest in various articulations throughout the body, the spinal region, particularly the cervical spine, frequently serves as a prevalent site of occurrence [3]. Hence, in TCM diagnosis, the specific location of manifestation is referred to as joint subluxation of the site, with atlantoaxial joint subluxation (AJS) being a prevalent clinical condition. Meanwhile, AJS is also considered a type of cervical spondylosis. The primary visual indication of AJS is an asymmetrical gap observed between the lateral mass of the atlantoaxial joint and the odontoid process on cervical spine X-ray or computed tomography (CT) scans, along with misalignment of the axis of the spinous process relative to the midline of the spinous process [4].

The atlantoaxial joint, situated in the human cervical spine, functions as a relatively autonomous unit. Within this joint, the atlas assumes the role of the central axis for neck motion, contributing to over 40% of the rotational movement of the cervical spine. Conversely, the axis serves as the focal point for stress in the upper cervical spine and functions as a primary attachment site for the neck’s short muscles and ligaments [5]. In instances where the atlantoaxial joint experiences a pathological state known as “joint subluxation,” it can lead to the entrapment of the synovial membrane and joint capsule, resulting in localized swelling and pain in the cervical spine, impaired joint movement, and stimulation of the corresponding regions of the nerves, blood vessels, and receptors, which can cause symptoms such as vertigo and pain [6]. For a long time, AJS has been commonly referred to as atlantoaxial joint instability, atlantoaxial joint disorder, etc [7,8]. The clinical manifestations are mainly neck pain accompanied by dizziness, headache, and cervical limited mobility, and thus, clinicians previously used to diagnose patients as having cervical vertigo [9,10].

Cervical manipulation is a frequently employed approach in the management of cervical spondylosis, with cervical rotation manipulation or small joint mobilization being the most prominent technique [11,12]. This method involves the rotation of the subject’s head along the longitudinal axis of the cervical spine, resulting in an audible “clicking” sound from the cervical vertebra joint. The clinical use of this technique has garnered significant attention and has been supported by empirical evidence demonstrating its efficacy and safety through various studies. Studies have demonstrated that this technique has the potential to ameliorate the pathological condition of joint subluxation, restore bones and joints to their typical physiological and anatomical alignments, and re-establish dynamic and static mechanical equilibrium in the cervical vertebra joint, thereby accomplishing therapeutic objectives [13-15]. Nevertheless, variations in clinical effectiveness arise due to divergent approaches to manipulation and the focus of the manipulation on the atlantoaxial joint, a crucial anatomical region within the cervical spine, and the safety of manual therapy has always been a concern of clinicians [16,17].

Shi cervical rotational manipulation (SCRM) is a technique for treating AJS created by Shi’s Traumatology TCM Academic School, which is the largest traumatology school of TCM in Shanghai. The technical attributes of SCRM encompass the swift application of stress, which belongs to the high velocity and low amplitude (HVLA) technique. In our prior investigation, we formulated a standard operating procedure (SOP) for SCRM, which will be explained subsequently [18]. Our research team quantified objective metrics and kinematic and kinetic parameters pertaining to the technique, considering the viewpoints of both recipients and practitioners [19]. Furthermore, we assessed the safety of the technique through finite element analysis and provided preliminary insights into its biomechanical mechanism of action [20]. Regarding the clinical assessment of the immediate efficacy of SCRM, a 50% reduction in visual analog scale (VAS) scores was observed immediately following the procedure. Additionally, approximately 70% to 80% of participants reported subjective improvements such as a sensation of a lighter head, a relaxed neck, and brighter eyes [18]. These findings suggest the significant immediate efficacy of the SCRM technique. In our subsequent short-term and medium-term clinical follow-up study in which we applied SCRM to treat patients with cervical spondylotic radiculopathy, the intervention had an efficacy rate of 94.74%. Furthermore, the overall efficacy rate at 3 months was 89.47%, with no notable discomfort or adverse events reported [18]. Nonetheless, there is still a lack of standard randomized controlled studies on SCRM, and there is limited evidence-based medical information on the efficacy and safety of SCRM. Consequently, the aim of this randomized controlled trial is to assess the efficacy and safety of SCRM in patients with AJS.

## Methods

### Study Design

**Figure 1:**
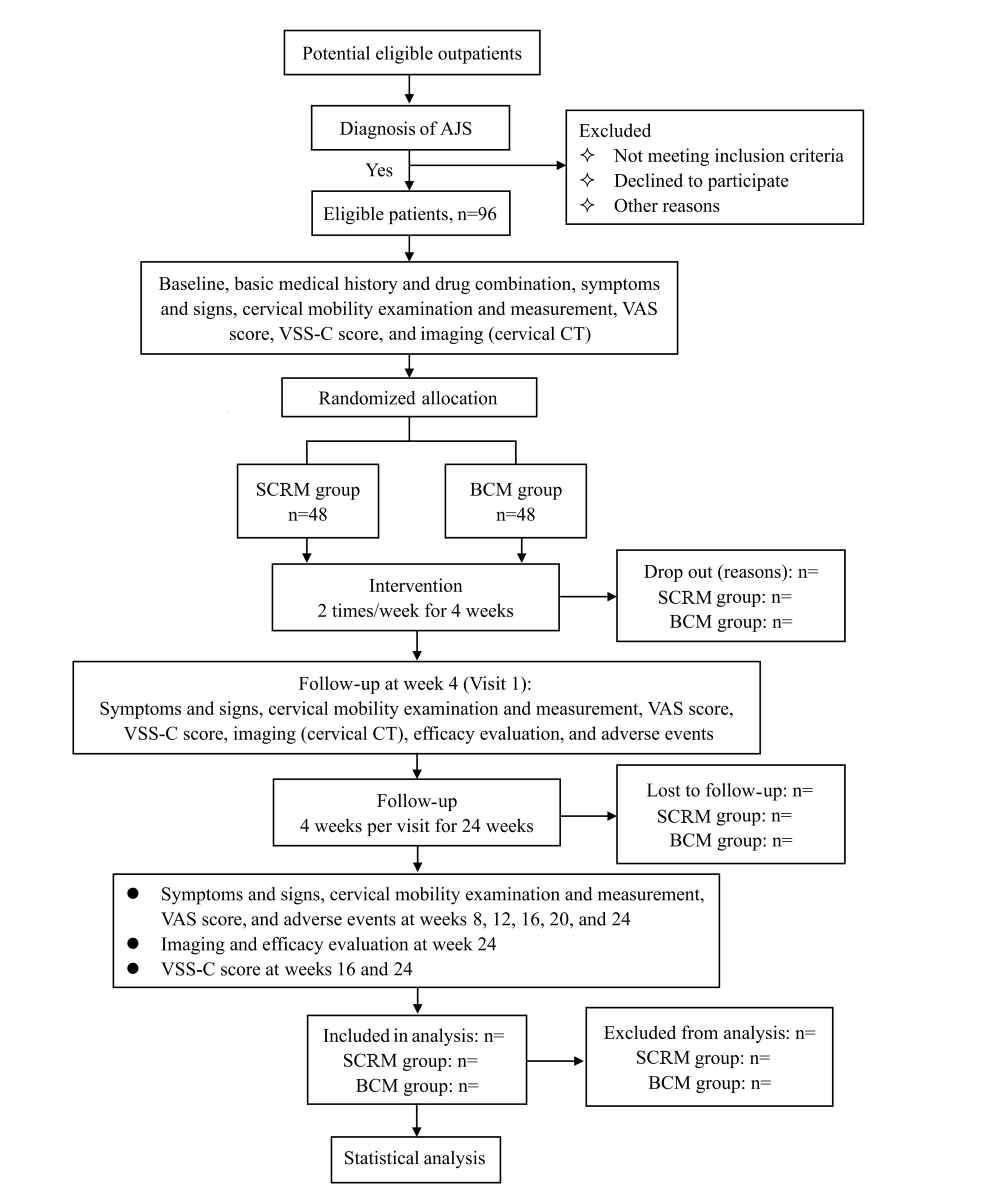
Flow diagram of participants. AJS: atlantoaxial joint subluxation; BCM: basic cervical manipulation; CT: computed tomography; SCRM: Shi cervical rotational manipulation; VAS: visual analog scale; VSS-C: Vertigo Symptoms Scale-Chinese version.

This study will be a single-center, prospective, randomized controlled clinical trial to evaluate the effectiveness and safety of SCRM in patients with AJS. We have used the SPIRIT (Standard Protocol Items: Recommendations for Interventional Trials) reporting guidelines to introduce this protocol [21]. A total of 93 participants with AJS from outpatient and inpatient clinics will be recruited and randomly allocated to either the SCRM group or the basic cervical manipulation (BCM) group. Screening (visit 0) will be performed within 3 days prior to enrollment to assess eligibility and collect baseline data and imaging data. Those participants who meet the inclusion criteria will be randomly assigned to the SCRM group or BCM group and receive the corresponding treatment of each group twice a week for 4 weeks. After the end of treatment (visit 1), all patients will undergo evaluations, including assessment of symptoms and signs, cervical mobility examination and measurement, assessment of VAS scores and Vertigo Symptoms Scale-Chinese Version (VSS-C) scores, imaging, efficacy evaluation, and assessment of adverse events. Then, all patients will be followed up every 4 weeks until the end of the study at 24 weeks. Additionally, imaging and efficacy evaluations will be conducted at week 4 (visit 1) and week 24 (visit 6), and assessment of the VSS-C score will be conducted at week 4 (visit 1), week 16 (visit 4), and week 24 (visit 6). The flowchart schedule is shown in Table 1, and the flow diagram of participants is shown in Figure 1.

**Table 1:** Flowchart schedule.

Assessment	Time window						
	Visit 0 (screening stage; –3 to 0 days)	Visit 1 (week 4)	Visit 2 (week 8)	Visit 3 (week 12)	Visit 4 (week 16)	Visit 5 (week 20)	Visit 6 (week 24)
Baseline information	Yes	No	No	No	No	No	No
Basic medical history and drug combination	Yes	No	No	No	No	No	No
Inclusion and exclusion criteria	Yes	No	No	No	No	No	No
Informed consent	Yes	No	No	No	No	No	No
Randomization allocation	Yes	No	No	No	No	No	No
Symptoms and signs	Yes	Yes	Yes	Yes	Yes	Yes	Yes
Cervical mobility examination and measurement	Yes	Yes	Yes	Yes	Yes	Yes	Yes
VAS^a^ score	Yes	Yes	Yes	Yes	Yes	Yes	Yes
VSS-C^b^ score	Yes	Yes	No	No	Yes	No	Yes
Imaging (CT^c^ or X-ray)	Yes	Yes	No	No	No	No	Yes
Efficacy evaluation	No	Yes	No	No	No	No	Yes
Adverse events	No	Yes	Yes	Yes	Yes	Yes	Yes

^a^VAS: visual analog scale.

^b^VSS-C: Vertigo Symptoms Scale-Chinese Version.

^c^CT: computed tomography.

The Shanghai Baoshan Hospital of Integrated Traditional Chinese and Western Medicine will be responsible for the recruitment, screening, and intervention of all participants and the assessment of all outcomes. Management of the randomization sequence and data analyses will be carried out by the Institute of Traumatology, Shi’s Center of Orthopedics and Traumatology, Shuguang Hospital Affiliated to Shanghai University of Traditional Chinese Medicine.

### Participants

Patients who are aged between 18 and 70 years with AJS will be included in the study. The screening, inclusion, and exclusion procedures for all patients; the notification and signing of informed consent; and the documentation of each follow-up visit will be meticulously recorded on an individual basis.

### Diagnostic Criteria

The diagnosis of AJS is based on the Diagnostic Criteria for Traditional Chinese Medicine and the International Classification of Diseases 11th Revision (ICD-11), and several aspects are considered. First, the patient has a history of neck strain or injury and has a long-term habit of working or living with a low head. Second, the clinical manifestations are mainly vertigo, dizziness, headache, and soreness in the neck. Third, the cervical movement is mainly limited to the left-right rotation of the atlantoaxial joint. When performing palpation, the muscle tension around the atlantoaxial joint increases or small blocky or strip-shaped muscle nodules are felt, which may induce or exacerbate dizziness or vertigo. Fourth, cervical X-ray or CT shows an asymmetrical gap between the lateral mass of the atlantoaxial joint and the odontoid process, with a difference of more than 2 mm, along with the misalignment of the axis of the spinous process relative to the midline of the spinous process.

### Inclusion Criteria

The inclusion criteria are as follows: (1) meeting the diagnostic criteria of AJS; (2) male or female sex and age of 18 to 70 years; (3) recurrent clinical symptoms, such as neck pain, vertigo, dizziness, and headache, for less than 4 weeks, which cannot be relieved; and (4) voluntary participation in this trial and signing of the informed consent document.

### Exclusion Criteria

The exclusion criteria are as follows: (1) cervical spondylosis of the nerve root type or spinal cord type and severe spinal stenosis; (2) a history of spinal surgery or severe spinal trauma; (3) a history of congenital variation or malformation of an unstable cervical vertebral structure; (4) dizziness or vertigo caused by other diseases diagnosed by specialized examinations such as those performed by cardiovascular, otolaryngology, and neurology departments; (5) imaging findings, including spinal infection, fracture, tumor, tuberculosis, severe spinal deformity, severe osteoporosis, ankylosing spondylitis, and osteitis deformans; (6) severe primary diseases of the endocrine system, cardiovascular system, and autoimmune system, and tumor or mental illness; (7) pregnancy and breastfeeding; and (8) no acceptance of cervical manipulation therapy.

### Withdrawal Criteria

The withdrawal criteria are as follows: (1) severe or intolerable adverse reactions during the observation process; (2) persistent or progressive symptoms and no suitability for participation in the trial; (3) risk of health damage (such as sudden serious complications); and (4) voluntary withdrawal or interview miss for other reasons.

### Sample Size

The sample size calculation is based on our previous clinical research in patients with cervical spondylosis of the nerve root type, using the same primary outcome instrument. The efficiency of SCRM is 89.5%, while that of BCM is 54.6%. The sample size calculation formula is as follows:



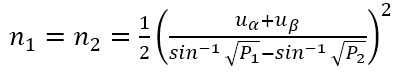



The α is .05 and 1–β is .90. According to estimations, 48 participants per group are needed considering that this is a long-term follow-up that is expected to have a dropout rate of no greater than 20%.

### Randomization and Allocation

The randomization schedule will be prepared by the Institute of Traumatology, Shi’s Center of Orthopedics and Traumatology, Shuguang Hospital Affiliated to Shanghai University of Traditional Chinese Medicine. The specific randomization number lists will be computer-generated using IBM SPSS 22.0 (IBM Corp) and concealed from the screeners, assessors, and patients by a specialized staff member who is not involved in the study. Within a set of randomly generated numbers, odd numbers will be attributed to the SCRM group, while even numbers will be attributed to the BCM group. Subsequently, all numbers and their respective group assignments will be documented on a card and securely sealed within opaque envelopes. These envelopes will be labeled with sequential numbers corresponding to the patients and entrusted to the screeners who bear the responsibility of participant screening. Once a patient is successfully enrolled, provides written informed consent, completes the baseline measurements, and is confirmed as an eligible participant, the sealed envelope will be transferred to the manual therapist. The manual therapist will unseal the envelope and administer the intervention (SCRM or BCM) based on the instructions provided on the card. The allocation list containing sensitive information will be kept confidential by specialized personnel who are not involved in participant recruitment or outcome assessment. This information will not be disclosed to the data analysts or outcome assessors.

### Blinding

Participants will be unaware of their group assignment and will remain unaware of receiving SCRM or BCM treatment. The outcome assessors will also be unaware of the randomization allocation and will not be involved in the intervention process. The manual therapists will not be able to be blinded owing to their involvement in performing the interventional protocols. However, they will not participate in the outcome measurements or statistical analyses and will be instructed to refrain from disclosing treatment details to the outcome assessors or participants. The statistician will remain blinded to the group allocation until the completion of the statistical analyses.

### Interventions

The techniques used in this study are SCRM in the experimental group and BCM in the control group. The two techniques are commonly used in clinical treatment and have established SOPs in Shanghai. All patients will receive treatment twice a week, with a 2-day interval between sessions, for a duration of 4 weeks. In addition, there will be a relaxation therapy step before the 2 techniques, after which the SCRM or BCM technique will be performed. The relaxation therapy will be performed as follows:

1. The patient assumes a seated position, while the manipulation therapist positions themselves behind one side of the patient’s body.
2. One hand of the therapist provides support to the patient’s forehead, while the other hand employs pressing, pulling, and kneading techniques on both sides of the patient’s cervical spine, as well as the trapezius, sternocleidomastoid, and levator scapulae muscles, repeating these actions 3 times.
3. Subsequently, the therapist applies pushing techniques along the line extending from the 7th cervical spine to the acromion, repeating this action 3 times.

The entire relaxation therapy session lasts approximately 10 minutes.

### SCRM Technique

**Figure 2:**
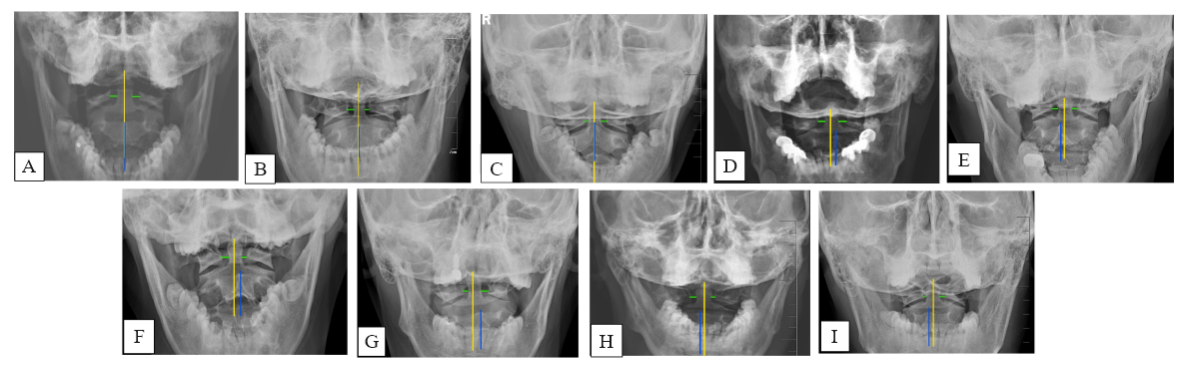
Comprehensive classification of atlantoaxial joint subluxation using X-ray. The green line indicates the distance between the lateral mass of the atlantoaxial joint and the odontoid process. The blue line indicates the axis line of the C2 spinous process. The yellow line indicates the midline of the spinous process. (A) Normal atlantoaxial joint; (B) Type A; (C) Type B; (D) Type C; (E) Type D; (F) Type AC; (G) Type AD; (H) Type BC; (I) Type BD.

The primary technological approach in SCRM involves applying pressure with the thumb on the spinous process, transverse process, or pedicle of the patient’s axis while simultaneously rotating the cervical spine in an effort to restore it to its original alignment. AJS is categorized into 8 distinct techniques, which are determined by the specific imaging characteristics of the atlantoaxial joint. Figure 2 shows the comprehensive classification of AJS according to X-ray findings. Each technique within these categories employs different SCRM methods. When there is a simple asymmetrical gap between the lateral mass of the atlantoaxial joint and the odontoid process, the spinous process of the axis remains aligned with the midline of the spinous process, yet the discrepancy in distance between the lateral mass of the left and right atlas and the dentate process is evident. If the left block displays a 2-mm greater distance compared with the right block, it falls under type A (left oblique type). Conversely, if the right block displays a 2-mm greater distance compared with the left block, it is classified as type B (right oblique type). When there is a simple misalignment of the axis spinous process relative to the midline of the spinous process, the distance between the lateral masses of the left and right atlas and the dentate process is either equal or exhibits a difference within a 2-mm range. However, the spinous process of the axis deviates from the midline of the spinal axis. If the spinous process of the axis is located at the left of the midline, it is classified as type C (left rotation type). Conversely, if the spinous process of the axis is located at the right of the midline, it is categorized as type D (right rotation type). The types can mix as follows: when type A merges with type C, it is referred to as type AC; when type A merges with type D, it is referred to as type AD; when type B merges with type C, it is referred to as type BC; and when type B merges with type D, it is referred to as type BD. The SOPs of SCRM according to the different types are presented below [18].

For type A, the steps are as follows. First, the patient assumes a seated position, while the medical therapist stands behind the patient on the left side. The therapist’s left hand supports the patient’s forehead, specifically positioning the right thumb at the left transverse process of the patient’s axis. The remaining 4 fingers of the therapist’s right hand rest gently on the right side of the patient’s neck, resulting in a forward flexion of the patient’s neck by approximately 10°. Second, the therapist subsequently alters the position of their left hand, placing it on the patient’s right chin nodule, while maintaining the unchanged posture of the right hand. This adjustment allows the therapist to rotate the patient’s neck to its maximum range of active movement. Third, the therapist’s left hand persists in applying force to achieve the maximum range of passive movement in rotating the patient’s neck, while the right thumb maintains a fixed position. The patient is instructed to relax their neck. Subsequently, the left hand momentarily intensifies the force of left rotation, while the right thumb applies pressure to the transverse process in a rightward direction. This maneuver often elicits an audible “click” sound emanating from the neck, accompanied by a subtle sliding sensation beneath the therapist’s right thumb, and the procedure is considered concluded.

For type B, the therapist stands behind the patient’s right side, and the therapist’s right hand supports the patient’s forehead, specifically positioning the left thumb at the right transverse process of the patient’s axis. The remaining steps are the same as those for type A, except that they are all performed in the opposite direction.

For type C, the operation steps are the same as those for type A, while the right thumb of the therapist is fixed at the left spinous process of the patient’s axis.

For type D, the operation steps are the same as those for type B, while the left thumb of the therapist is fixed at the right spinous process of the patient’s axis.

For type AC, the operation steps are the same as those for type A*,* while the right thumb of the therapist is fixed at the left pedicle of the patient’s axis.

For type AD, the first operation steps are the same as those for type A, and the second operation steps are the same as those for type D*.*

For type BC, the operation steps are the same as those for type B*,* while the left thumb of the therapist is fixed at the right pedicle of the patient’s axis.

For type BD, the first operation steps are the same as those for type B, and the second operation steps are the same as those for type D*.*

### BCM Technique

The BCM technique does not differ depending on the type of AJS. The SOP of BCM for patients with AJS (all types) is as follows [18]. First, the patient assumes a seated position, and the manipulation therapist stands behind the patient’s left side. Second, the therapist manipulates the patient’s jaw by flexing their left elbow and applies pressure to the occipital area with their right hand, which is supporting the occipital bone with the right palm and supporting the other 4 fingers. Third, the therapist gradually rotates the patient’s head to the left until reaching the active limit position and then instructs the patient to relax their neck while continuing to rotate the head to the passive limit position using both hands. During this process, a momentary force is exerted to further facilitate the left rotation of the patient’s head, resulting in audible clicking sounds emanating from the neck. Fourth, the therapist performs the 3 steps on the right side of the patient. It is important to note that the therapist can freely select one side (left or right) of the patient for the procedure.

### Follow-Up Period

Follow-up assessments with questionnaires will be conducted. During the 24-week unsupervised follow-up period, no participants will undergo special therapy, with the exception of routine cervical care.

It is important to acknowledge that every subsequent patient will schedule an appointment in advance via telephone and visit the outpatient department. Subsequently, the outcome assessors will aid patients in completing the questionnaire, will conduct and document physical examinations, and will administer cervical CT scans.

The outcome assessors will establish an electronic information group, inviting all participants to join. Throughout the follow-up period, any adverse events or physical discomfort experienced in any circumstance will be promptly reported within the information group.

### Outcome Measurement

The following baseline descriptive data will be obtained using questionnaires: age, gender, marital status, education level, occupation, basic medical history, and drug combination. A summary of all measures and follow-up time points in this trial is shown in Table 1.

#### Primary Outcome

##### Efficacy Rate

The clinical symptoms of all subjects will be recorded and evaluated using the numerical rating scale (NRS), which is composed of a horizontal line having a length from 0 to 10 cm, with “0” indicating “normal” and “10” indicating “unbearable.” We will refer to the efficacy evaluation standards in the “Diagnostic and Therapeutic Efficacy Standards for Traditional Chinese Medicine Diseases” issued by the China Administration of Traditional Chinese Medicine in 1994.

We define clinical cure as a decrease of more than 80% in the NRS score for clinical symptoms and the ability to live and work normally. We define clinical improvement as a decrease of 20% to 80% in the NRS score for clinical symptoms. We define clinical cureless as a decrease of less than 20% in the NRS score for clinical symptoms. We define clinical recurrence as the reappearance of the original symptoms after treatment, with the degree being equivalent to or greater than that before treatment.

All patients will be followed up, and the efficacy rate will be calculated at visit 1 after the intervention. The efficacy rate and clinical recurrence rate will be calculated at visit 6. The calculation formulas are as follows:

Efficacy rate = ([number of clinical cure cases + number of clinical improvement cases – number of clinical cureless cases] / total number of patients) 100

Clinical recurrence rate = (number of clinical cureless cases / [number of clinical cure cases + number of clinical improvement cases]) 100

#### Secondary Outcomes

##### Severity of Neck Pain (VAS)

The VAS score will be used to evaluate the severity of neck pain in all participants [22]. The VAS score sheet consists of a horizontal 100-mm line with “painless” and “extremely painful” end points. Patients select a point on the line segment based on their own pain perception and draw a vertical line through that point to express the intensity of pain.

##### Severity of Vertigo (VSS-C)

The VSS-C scale has been used in many studies to evaluate the severity of vertigo and has shown high reliability and validity [23]. The VSS-C is a self-assessment scale consisting of 22 questions, with a score of 0-4 for each question. A total score of 0-33 points indicates mild vertigo, 34-67 points indicates moderate vertigo, 68-101 points indicates severe vertigo, and 102-136 points indicates extremely severe vertigo.

##### Imaging Changes of Cervical CT

All participants will undergo cervical CT examination at visits 0, 1, and 6. Two radiologists will measure the distance from the lateral mass of the atlantoaxial joint to the dentate process and the distance from the axis to the midline of the spinous process. Each radiologist will measure each image 3 times, and the average of the 6 outcomes will be taken as the final result. The improvement rate will then be calculated based on the results. The calculation formula is as follows:

Improvement rate = ([distance at pretreatment – distance after treatment] / distance at pretreatment) 100

##### Cervical Mobility

A cervical inclinometer will be used to measure cervical mobility. The outcome assessor will measure and record the cervical angles of the patient in 6 directions: flexion, extension, left rotation, right rotation, left lateral bending, and right lateral bending [24]. The outcome assessor will take 3 measurements in each direction, and the average of the 3 values will be used as the final result.

### Adverse Events

Any adverse events, including symptoms, signs, and physical or laboratory examination abnormalities, will be carefully evaluated and recorded. Researchers will analyze the causes of these adverse events, make judgments, and track and record them in a timely manner. Once an adverse event occurs, doctors will provide the corresponding treatment to the patient for free. All adverse events will be judged for their characteristics, severity, and potential relationship to the study treatment. The correlation between adverse events and the study treatment is divided into 5 levels: definitely related, probably related, possibly related, possibly unrelated, and definitely unrelated. Based on the judgment and severity of adverse events, the primary investigator and ethics committee will be informed immediately to decide whether to discontinue observation, retain the medical records of participants who withdraw from the experiment, conduct a full data set analysis of the efficacy and adverse reactions, and fill in the experiment conclusion and the reason for case withdrawal.

### Data Collection and Management

An electronic information data collection system for our research has been created by the Institute of Traumatology, Shi’s Center of Orthopedics and Traumatology. All data will be electronic, double-checked, and backed up by 2 research assistants and entered into the system for mutual verification and correction of errors. Once all electronic data are verified against the original data, the data will be locked, and any modifications and their reasons will leave a trace in the data backend.

### Statistical Analysis

The main objective of this study is to evaluate the clinical effectiveness of SCRM therapy in patients with AJS. Additionally, secondary analyses will be conducted to assess changes in VAS scores, VSS-C scores, imaging findings, and cervical mobility before and after treatment in patients with AJS. Analyses will be performed using IBM SPSS 22.0. The measurement data will be expressed using mean differences (mean and standard deviation). The repeated measurement data will be analyzed using within-group variance analysis. Enumeration data will be presented as rates. The sample size needed for the Pearson chi-square test to compare rates between the 2 groups before and after treatment will be determined using the normal approximation algorithm and a 1-sided test. This study employs the superiority test that uses a 1-sided test level of α=.05. A *P* value of ≤.05 signifies the rejection of H0, thereby establishing the superiority of the SCRM group over the BCM group. Conversely, a *P* value >.05 indicates that a definitive conclusion regarding superiority cannot be drawn at this stage. The safety analysis will employ the chi-square test to compare the incidence of adverse reactions between the 2 groups. In instances where the data fail to meet the requirements of the chi-square test, the Fisher exact probability method will be employed.

### Ethical Considerations

The study procedures have been reviewed and approved by the Institutional Review Board of Shanghai Baoshan Hospital of Integrated Traditional Chinese and Western Medicine (approval number: 202218), and protocol version 1.0/20221201 is currently active. The full trial has been registered through the Chinese Clinical Trial Registry (identifier: ChiCTR2300068510). Written informed consent will be obtained from all participants before enrolment in the study. Any modifications to the protocol related to deviation of the study aim, study design, patient population, or study procedures will not be implemented until the formal amendment to the protocol is approved by the Institutional Review Board.

## Results

This paper presents an overview of the reasoning and structure of a prospective randomized controlled trial with the objective of investigating the clinical efficacy and safety of SCRM in patients with AJS by assessing improvements in clinical symptoms, neck pain severity, and vertigo severity and evaluating changes in cervical imaging findings. Recruitment was started in March 2023. By the end of May 2024, 76 patients were included in this project. The last follow-up data are predicted to be collected by the end of February 2025. The findings of this study are expected to be submitted for publication in late 2025.

## Discussion

### Findings and Strengths

Globally, manual therapy is considered one of the most crucial clinical therapies for the treatment of cervical spondylosis, and approaches, such as spinal manipulation, chiropractic adjustment, the Mulligan technique, small joint adjustment, and the HVLA technique, are widely used [25-27]. In the context of China, doctors employ diverse techniques in various regions and schools, each possessing distinct technical attributes. In Shanghai, China, the most prevalent technique employed is SCRM, which has demonstrated satisfactory therapeutic outcomes [28]. However, there exists a dearth of evidence-based medical information regarding its efficacy and safety. Consequently, this study aims to examine the effectiveness and safety of SCRM in treating patients with AJS through a prospective randomized controlled trial.

The optimal sample size has been accurately determined based on the findings of our prior research to guarantee sufficient test performance [18]. Subsequently, randomization and blinding procedures will be fully implemented to ensure the study’s classification as a randomized controlled trial. Additionally, a statistical analysis employing a superiority test will be conducted to ascertain that all patients with AJS can receive authentic clinical treatment. As Shi’s Orthopedics and Traumatology Academic School is well known throughout China, with a history of 160 years, it is officially recognized by China’s Health Care Administration, and Shanghai Baoshan Hospital is a branch of Shi’s Orthopedics and Traumatology Academic School. We ensure that the interventions (SCRM and BCM) will be performed by a fixed chief physician with more than 10 years of clinical experience. Moreover, proficient research assistants will be assigned to carry out screening, selection, and follow-up, possessing a comprehensive understanding of the research process for all participants. These measures will greatly contribute to enhancing patient adherence and mitigating the likelihood of attrition in this study. In terms of evaluation metrics, we will employ appropriate standards and develop meticulous professional scales as the primary outcome evaluation indicators to prevent subjective and ambiguous classification assessments, thereby minimizing data distortion and bias. Lastly, we have developed a dedicated electronic information data collection system for this study, guaranteeing the precision, novelty, and security of the data. This study aims to examine the clinical effectiveness and safety of SCRM in patients with AJS by assessing improvements in clinical symptoms, neck pain severity, and vertigo severity and evaluating changes in cervical imaging findings. If successful, this investigation will yield dependable evidence on the efficacy and safety of SCRM in individuals with AJS.

### Limitations

For classification, AJS has been divided into 8 types. For the different types, the SCRM techniques are different, while the BCM techniques are the same. As the incidence rate of AJS is not high, it may be difficult to recruit patients with each type of AJS, who meet the statistical requirements, in a limited time. Therefore, we will not separate the different AJS types, but instead combine them to observe the difference in efficacy between SCRM and BCM. In the future, we will further extend our project timeline and recruit as many subjects as possible for each type of AJS, so that efficacy analysis can be conducted for different types of AJS.

## Abbreviations

AJS: atlantoaxial joint subluxation
BCM: basic cervical manipulation
CT: computed tomography
HVLA: high velocity and low amplitude
NRS: numerical rating scale
SCRM: Shi cervical rotational manipulation
SOP: standard operating procedure
TCM: traditional Chinese medicine
VAS: visual analog scale
VSS-C: Vertigo Symptoms Scale-Chinese Version
